# Individuals with Tinnitus Report More Positive Experiences following Internet-Based Cognitive Behavioral Therapy

**DOI:** 10.3390/clinpract14040130

**Published:** 2024-08-21

**Authors:** Vinaya Manchaiah, Eldré W. Beukes, Gerhard Andersson, Emily Bateman, De Wet Swanepoel, Kristin Uhler, Vinay

**Affiliations:** 1 Department of Otolaryngology-Head and Neck Surgery, University of Colorado School of Medicine, Aurora, CO 80045, USA; bateman.emily9@gmail.com (E.B.); dewet.swanepoel@up.ac.za (D.W.S.); 2 UCHealth Hearing and Balance, University of Colorado Hospital, Aurora, CO 80045, USA; 3 Virtual Hearing Lab, Collaborative Initiative between University of Colorado School of Medicine and University of Pretoria, Aurora, CO 80045, USA; eldre.beukes@aru.ac.uk; 4 Department of Speech-Language Pathology and Audiology, University of Pretoria, Pretoria 0001, South Africa; 5 Department of Speech and Hearing, School of Allied Health Sciences, Manipal University, Manipal 576104, India; 6 Vision and Hearing Sciences Research Group, School of Psychology and Sports Sciences, Anglia Ruskin University, Cambridge CB1 1PT, UK; 7 Department of Behavioral Sciences and Learning, Department of Biomedical and Clinical Sciences, Linköping University, 582 25 Linköping, Sweden; gerhard.andersson@liu.se; 8 Department of Clinical Neuroscience, Division of Psychiatry, Karolinska Institute, 171 77 Stockholm, Sweden; 9 Department of Audiology, Children’s Hospital Colorado, Aurora, CO 80045, USA; kristin.uhler@cuanschutz.edu; 10 Department of Physical Medicine and Rehabilitation, University of Colorado School of Medicine, Aurora, CO 80045, USA; 11 Audiology Group, Department of Neuromedicine and Movement Sciences, Norwegian University of Science and Technology, 7030 Trondheim, Norway; vinay.nagaraj@ntnu.no

**Keywords:** tinnitus, chronic tinnitus, positive experiences, cognitive behavioral therapy, outcome measure

## Abstract

Background: This study aimed to examine whether individuals with chronic tinnitus report more positive experiences following internet-based cognitive behavioral therapy (CBT). Methods: A mixed-methods design was used, nested in clinical trials evaluating internet interventions for tinnitus. Participants (*n* = 164) completed online questionnaires (both structured and open-ended) providing demographic information as well as health variables (e.g., tinnitus distress, anxiety, depression, insomnia). An open-ended question listing positive effects or outcomes related to having tinnitus was also included. Responses to the open-ended questions were analyzed using qualitative content analysis. Results: Of the 164 eligible participants, 32.3% (*n* = 53) provided at least 1 positive experience both at pre- and post-intervention, with 9.1% (*n* = 19) providing positive experiences only at pre-intervention, 49 (29.9%) providing positive experiences only at post-intervention, and 28.7% (*n* = 47) of the participants did not provide any positive experiences on either measurement occasion. Significantly more positive experiences were reported following the intervention in the overall sample (*p* < 0.0001, paired sample *t*-test). In addition, participants who reported positive experiences in both pre- and post-intervention also reported more positive experiences following intervention (*p* = 0.008, paired sample *t*-test). Conclusions: Internet-based CBT can help individuals with tinnitus to think more positively by changing unhelpful thought patterns. Open-ended questions can supplement structured questionnaires to measure treatment outcomes.

## 1. Introduction

Individuals with tinnitus experience perception of sound(s) in the absence of any external sound stimulus. Tinnitus is a common chronic condition in adults, affecting nearly 15% of the population around the world [1]. While a majority of individuals with tinnitus adapt well and learn different ways to cope with the condition, some of the individuals with severe tinnitus may develop maladaptive strategies and experience many negative consequences such as difficulty focusing on a task, difficulty sleeping, anxiety, depression, and reduced health-related quality of life [2,3]. For this reason, many individuals with tinnitus may need an intervention to manage their condition and to live well with their tinnitus.

Tinnitus can be of various types [4]. For instance, if no clear cause can be found, it is referred to as primary tinnitus. On the other hand, if there is an identifiable cause (e.g., ear infection, ear drum perforation, vascular disease), it is referred to as secondary tinnitus. Also, chronic tinnitus is referred to as experiencing these sounds for three months or longer. 

Currently, there is no known cure for tinnitus, with a few exceptions when an otological condition is causing tinnitus (e.g., ear infection). There are, however, several management strategies that can help those individuals who experience negative consequences. These can be broadly grouped into four groups. First, psychological interventions such as cognitive behavioral therapy (CBT). Second, sound therapy-based interventions such as hearing aids, masking devices, or neuromodulation (i.e., training the brain to ignore tinnitus sound through delivery of sound, electricity, or other stimuli). It is noteworthy that hearing aid intervention is suggested for individuals with tinnitus who have an accompanying hearing loss. Third, a combination of psychological and sound-therapy-based interventions such as progressive tinnitus management (PTM) or Tinnitus Retraining Therapy (TRT). Fourth, alternative/complementary medicine treatments such as herbal therapies, etc. Out of these management strategies, CBT intervention has the best research support for reducing tinnitus distress [5]. CBT is a form of psychotherapy and aims to improve the quality of life in individuals with tinnitus through principles of habituation, cognitive restructuring, and the development of coping skills [6]. Relaxation strategies as well as thought modification involve skills to handle dysfunctional cognitions in relation to tinnitus (such as catastrophizing) [7]. While clinical trials on CBT have reported a range of positive outcomes in terms of reduced tinnitus distress, insomnia, anxiety, and depression, the extent to which CBT strategies increase positive thoughts and experiences in individuals with tinnitus has not been reported.

A sub-field of psychology studying positive experiences related to chronic health conditions and disabilities is referred to as positive psychology. Positive psychology focuses on wellbeing with the idea that individuals with chronic conditions may benefit from creating a context for wellbeing in addition to symptom reduction [8]. Several studies have examined the effect of using a positive psychology approach to building psychological interventions for various conditions and have reported positive outcomes [9,10]. In addition, during the last decade, numerous studies have examined positive experiences related to hearing and balance conditions such as hearing loss, tinnitus, and Ménière’s disease (for review see [11]. Three separate cross-sectional studies conducted in Finland [12], the United Kingdom [13], and the United States [14] have examined and reported positive experiences related to tinnitus elicited from an open-ended question. The common themes tend to be about outlook on life (e.g., I am grateful for my life), personal development (e.g., I am more aware of what I can handle), treatment-related benefits (e.g., I am protecting my ears by wearing ear plugs), coping strategies (e.g., learned how to manage stress through relaxation exercises), support received (e.g., compassion from others with tinnitus), and disease-specific aspects (e.g., I think it may actually help lull me to sleep).

Despite the progress in adopting positive psychology approaches to hearing health, much of the published literature focuses solely on the natural course of disease and open-ended questions on how individuals with hearing and balance problems identify and report positive experiences. The studies do provide some understanding of acceptance and coping. We are, however, not aware of any studies examining the effects of psychological interventions in promoting positive psychology within the field of hearing and also more specifically to tinnitus populations. The current study therefore examined if individuals with tinnitus report more positive experiences to an open-ended question following an internet-based CBT (ICBT).

## 2. Methods

### 2.1. Study Design

This study used a pretest–posttest design and was nested in clinical trials (Clinical Trials.gov registration numbers NCT04004260, NCT04335812) that were aimed at evaluating the efficacy of ICBT for tinnitus [15,16,17]. Ethics approval was granted by the Institutional Review Board at Lamar University (IRB-FY17-209 and IRB-FY20-200). All participants completed informed consent prior to participating in the study. 

### 2.2. Data Collection

All participants (*n* = 164) who were enrolled in the ICBT clinical trials were asked to complete a series of questionnaires before and after the intervention through the online platform (i.e., iTerapi) that was used to administer ICBT. The inclusion (i.e., adults > 18 years, ability to read and type in English or Spanish, access to computer, having tinnitus for longer than 3 months, at least a mild severity of tinnitus) and exclusion criteria (i.e., having significant depression, psychiatric condition, pulsatile or objective tinnitus, currently undergoing other tinnitus therapies) are detailed in the clinical trial publications [15,16,17]. Chronic tinnitus was defined as experiencing tinnitus for at least 3 months or longer. These included (a) a demographic questionnaire, (b) several standardized structured questionnaires focusing on tinnitus distress (i.e., Tinnitus Functional Index; TFI), anxiety (i.e., Generalized Anxiety Disorder 7; GAD7), depression (i.e., Patient Health Questionnaire 9; PHQ9), insomnia (i.e., Insomnia Severity Index; ISI), and health-related quality of life (i.e., Euroqol EQ-5D-5L), and (b) open-ended questions. The current study was focused on analyzing the responses to an open-ended question focusing on positive experiences, which was worded as “Make a list of any positive effects or outcomes related to having tinnitus, list as many as you can think of”. Further details about the data collection have been provided in our clinical trial reporting [15,16,17]. The study sample was categorized into four groups based on their response patterns to the open-ended question: Group 1 (*n* = 53): both pre- and post-intervention; Group 2 (*n* = 15): only during pre-intervention; Group 3 (*n* = 49): only during post-intervention; and Group 4 (*n* = 47): no response in both pre- and post-intervention.

### 2.3. Data Analysis

The data analysis included a mixed methods approach. The demographic items and responses to structured questionnaires across groups were analyzed using descriptive and analytical statistics using the IBM SPSS software version 29. Responses to open-ended questions were coded using qualitative content analysis [18]. Only the responses of Group 1 were included in the qualitative analysis. Differences in the mean number of responses to open-ended questions in the overall sample as well as in groups that provided at least one response in both pre- and post-intervention were examined using the paired sample *t*-test as the data met the assumption of normality using the Shapiro–Wilk test (*p* > 0.05). 

## 3. Results

### 3.1. Participant Characteristics

One hundred and sixty-four individuals participating in ICBT clinical trials were included in the present study. Participants had a mean age of 58.44 years (SD: 11.54 years, mean duration of tinnitus in 13.91 years; 97 females and 67 males). Table 1 provides the participant characteristics, including responses to structured questionnaires across the four groups.

### 3.2. Number of Positive Experiences

**Figure 1:**
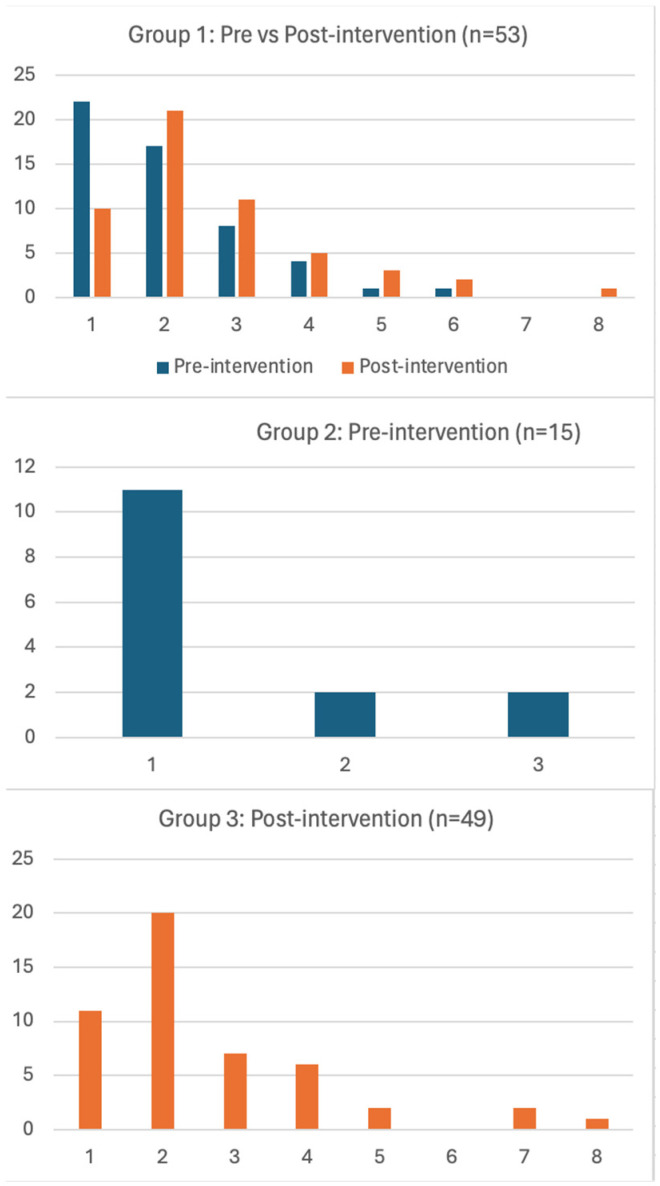
Number of positive experiences (x-axis) reported by individual study participants (y-axis) in different groups during pre- and post-intervention.

Figure 1 presents the number of positive experiences per participant across groups. Of the 163 participants, 54 (32.3%) provided at least one positive experience in both pre- and post-intervention (group 1), 15 (9.1%) provided positive experiences only during pre-intervention, 49 (29.9%) provided positive experiences only during post-intervention, and 47 (28.7%) participants did not provide any positive experience in both pre- and post-intervention. Table 2 presents the number of participants reporting positive experiences pre- and post-intervention in the overall sample as well as across the sub-groups. A total of 128 and 268 responses were reported in pre- and post-intervention, respectively, in the overall sample. More participants reported positive experiences following the intervention in the overall sample (pre-intervention mean = 0.78, post-intervention mean = 1.63, *p* < 0.0001). In addition, a total of 107 and 140 responses were reported in pre- and post-intervention, respectively, in Group 1 showing that participants who reported positive experiences in both pre- and post-intervention also reported more positive experiences following intervention (pre-intervention mean = 2.02; post-intervention mean = 2.6; *p* = 0.008).

### 3.3. Positive Experiences Related to Tinnitus

Table 3 presents the categories, sub-categories, frequencies, and an example of a meaning unit based on the qualitative content analysis of responses to open-ended questions on positive experiences. The analyses identified 6 categories and 23 sub-categories. The categories remained the same, for both pre- and post-intervention, although some variation in sub-categories was found. For instance, the sub-category learned new techniques and skills only emerged in post-intervention. The sub-categories empathy, resilience, self-care, self-improvement, spirituality, helping others, and new relationships were only found in pre-intervention responses. 

### 3.4. Outlook

This theme focused on change in outlook in terms of point of view or general attitude. Many participants reported that they realized they are not alone, developed appreciation for what they have and empathy towards others, and also developed self-awareness and resilience. This was the dominant theme in pre-intervention with 50 (46.7%) and 34 (24.3%) meaning units identified in pre- and post-intervention responses, respectively. 

### 3.5. Personal Development

Many individuals with tinnitus reported that experiencing chronic conditions such as tinnitus helped them reflect on their condition, which resulted in the realization of self-awareness of their capabilities as well as care for themselves, resulting in personal development. This theme was reported in 10 (9.3%) and 7 (5%) meaning units, respectively, in pre- and post-intervention. 

### 3.6. Coping

This theme included items on how individuals were coping with their condition. This included adaptation, making behavior changes, as well as finding spiritual reasons. The coping theme had 16 (15%) and 26 (18.6%) meaning units in pre- and post-interventions, respectively.

### 3.7. Support

Support from other is an important component in managing chronic conditions such as tinnitus. Many individuals with tinnitus reported that their condition helped them better understand their relationships as well as think about helping others. Many also sought help from local and national support groups, which helped them make new friends. This theme had 7 (6.5%) and 1 (0.7%) in pre- and post-interventions, respectively.

### 3.8. Treatment-Related

Managing tinnitus and in particular finding an evidence-based management for tinnitus is challenging. Hence, many individuals with tinnitus were appreciative of helpful treatments that resulted in positive experiences in terms of learning new techniques as well as making behavior changes. As expected, this was the dominant theme post-intervention. A total of 17 (16%) and 61 (43.5%) meaning units were identified in pre- and post-intervention responses, respectively.

### 3.9. Disease-Specific Experiences

Some individuals with tinnitus think they have positive experiences directly or indirectly as a result of living with this condition. This is a way for some individuals to accept the condition and learn to cope. This theme had 7 (6.5%) and 11 (7.9%) meaning units, respectively, in pre- and post-intervention responses. 

## 4. Discussion

Our findings suggest that more participants reported positive experiences following the intervention, which align with previous research demonstrating the efficacy of CBT in reducing tinnitus distress [5,6]. However, our study uniquely contributes to the literature by showing an increase in positive experiences, a novel outcome in the context of tinnitus management. The increase in positive experiences post-intervention could be attributed to the principles of cognitive restructuring and the development of coping skills inherent in CBT [9,10]. These elements help individuals reframe their perceptions of tinnitus, thereby fostering a more positive outlook.

In both research and healthcare service delivery, it is common to ask individuals with chronic conditions about the adverse effects of their illness, disease, or condition. This of course is key to understanding the extent to which the individual is impacted and to aid in the understanding of both severity and intervention strategies. However, this approach assumes that chronic conditions such as tinnitus could only have negative consequences. Recent disability models such as the World Health Organization’s International Classification of Functioning, Disability, and Health [19] recognize that the outcome of the disease and/or health conditions can be modified by contextual factors (i.e., environmental and personal factors). In this context, personal factors such as personality and temperament could make some individuals think alternatively and consider both positive and negative consequences. This small difference could indeed determine who may cope and manage well with their chronic condition versus those who do not. Nevertheless, an even more important question is if the interventions we provide could change the thought patterns of individuals with chronic conditions such as tinnitus and make them think more positively. The current study provides some preliminary data to support this idea. 

During the pre-intervention, outlook was the dominant theme (39% meaning units), suggesting that individuals who report positive experiences are mainly related to their point of view or attitude. On the other hand, treatment-related benefits were the dominant theme of positive experiences post-intervention, suggesting individuals can indeed derive positive benefits. Nevertheless, it is important to recognize that many individuals with chronic conditions such as tinnitus continue to look for a cure or silencing their tinnitus [20]. However, in conditions where we do not have a known cure, considering evidence-based treatments such as CBT and making them accessible and affordable through the use of digital technologies should be a priority. The study results also support the idea of including positive questioning during case history and during treatment sessions, especially using open-ended questions. 

Although many of this study’s participants reported finding positive experiences, many individuals noted a small number of positive experiences. Additionally, some participants were unable to identify positive experiences. These results are similar to those previously reported (e.g., [13], suggesting potential improvement with modification of the intervention. For instance, adding information in the interventions highlighting the main themes surrounding the key positive experience themes, such as outlook, support, and personal development, would be beneficial. It may also be that tailoring the intervention for certain populations is required. Loughlin et al. (2024), for instance, identified that younger participants and those with lower hearing disability were more likely to report positive experiences, which also appears to be the case in the present research. Older adults may need more guidance and examples. 

### 4.1. Clinical Implications

Clinicians should consider incorporating open-ended questions into their assessment protocols to capture a broader range of patient experiences. This approach can complement standardized measures and provide a more comprehensive understanding of treatment outcomes. Moreover, integrating modules that focus on fostering positive experiences and resilience into CBT programs for tinnitus could enhance overall treatment efficacy. Clinicians might also benefit from training in positive psychology techniques to support this integration.

### 4.2. Study Limitations

This study has some limitations that need consideration. First, this study was nested in a clinical trial that had many outcome measures. This may have resulted in an additional burden for individuals and, as a result, participants may have provided limited data for an open-ended question that is optional, resulting in a potential response bias. Second, our literature review on positive experiences related to hearing and balance conditions found that only about 40–45% of individuals provide answers to open questions, and this can increase to a 90% response rate when using structured questionnaires [11]. For this reason, developing a structured positive experience questionnaire based on qualitative data for future studies could be helpful. The core outcome set (COS) based on the Core Outcome Measures in Tinnitus (COMiT) initiative suggests including an outcome measure on negative thoughts and beliefs in psychology-based tinnitus intervention [21]. We would argue that it may be helpful to also include a positive approach focusing on negative thoughts to help understand symptom resolution, whereas examining positive aspects helps understand well-being. 

## 5. Conclusions

Overall, this study highlights the potential of internet-based CBT to not only reduce tinnitus distress but also enhance positive experiences among individuals with tinnitus. Incorporating positive psychology into tinnitus management could provide a more holistic approach to improving patient outcomes.

## Figures and Tables

**Table 1:** Demographic details of study participants (*n* = 163).

Groups	Group 1 (*n* = 53; 32.3%): Participants Reporting Positive Experiences Both Pre- and Post-Intervention	Group 2 (*n* = 15; 9.1%): Participants Reporting Positive Experiences Only during Pre-Intervention	Group 3 (*n* = 49; 29.9%): Participants Reporting Positive Experiences Only during Post-Intervention	Group 4 (*n* = 47; 28.7%): Participants Not Reporting Positive Experiences in Both Pre- and Post-Intervention
Age in years [Mean (SD)]	56.2 (13.6)	55.0 (17.2)	59.2 (9.8)	60.0 (7.9)
Tinnitus duration in years [Mean (SD)]	12.7 (15.7)	19.2 (18.7)	12.8 (15.7)	14.8 (10.9)
Gender [*n* (%)]				
▪ Male	19 (35.8)	9 (60.0)	14 (28.6)	25 (53.2)
▪ Female	34 (64.2)	6 (40.0)	35 (71.4)	22 (46.8)
Education [*n* (%)]				
▪ High school	4 (7.5)	1 (6.7)	5 (10.2)	7 (14.9)
▪ Some college but no degree	10 (18.9)	6 (40.0)	12 (24.5)	25 (53.2)
▪ A university degree	39 (73.6)	8 (53.3)	32 (65.3)	15 (31.9)
Work [*n* (%)]				
▪ Entry-level or unskilled work	0	1 (6.7)	2 (4.1)	2 (4.3)
▪ Skilled or professional work	33 (62.3)	9 (60.0)	25 (51.0)	24 (51.1)
▪ Retired	18 (34.0)	4 (26.7)	21 (42.9)	17 (36.2)
▪ Not working	2 (3.8)	1 (6.7)	1 (2.0)	4 (8.5)
Tinnitus distress (TFI) [Mean (SD)]				
▪ Pre-intervention	52.9 (20.9)	54.7 (19.8)	55.5 (22.1)	52.0 (21.6)
▪ Post-intervention	25.0 (19.3)	49.0 (21.9)	25.8 (17.6)	40.9 (22.8)
Anxiety (GAD-7) [Mean (SD)]				
▪ Pre-intervention	6.0 (4.8)	6.7 (5.9)	7.8 (5.5)	6.5 (5.7)
▪ Post-intervention	3.8 (3.9)	7.3 (4.8)	4.0 (4.3)	5.1 (5.1)
Depression (PHQ) [Mean (SD)]				
▪ Pre-intervention	6.4 (4.9)	7.1 (6.8)	7.3 (5.2)	7.1 (5.6)
▪ Post-intervention	3.6 (3.7)	7.0 (4.9)	3.6 (3.3)	5.7 (5.5)
Insomnia (ISI) [Mean (SD)]				
▪ Pre-intervention	10.8 (5.8)	11.9 (7.8)	11.2 (6.5)	11.8 (6.3)
▪ Post-intervention	6.5 (4.9)	9.7 (5.8)	5.9 (5.1)	9.6 (6.5)
Health-related quality of life (EQ-5D-5L VAS) [Mean (SD)]				
▪ Pre-intervention	79.3 (10.9)	78.6 (12.6)	76.4 (15.2)	72.4 (17.9)
▪ Post-intervention	79.6 (13.7)	71.3 (18.4)	80.2 (15.1)	75.0 (17.9)

**Table 2:** Total number of positive experiences reported by overall sample and different groups during pre- and post-intervention (statistical significance tested at *p* < 0.05).

Groups	Pre-Intervention	Post-Intervention	Mean (SD)	Paired-Sample *t*-Test Results
Overall sample (*n* = 164)	128	268	Pre-intervention: 0.78 (1.2) Post-intervention: 1.63 (1.8)	*t*(163) = −6.0, *p* ≤ 0.0001
Group 1 (*n* = 54)	107	140	Pre-intervention: 2.02 (1.2) Post-intervention: 2.6 (1.5)	*t*(52) = −2.8, *p* = 0.008
Group 2 (*n* = 15)	21	0		
Group 3 (*n* = 49)	0	128		
Group 4 (*n* = 47)	0	0		

**Table 3:** Positive experiences reported for an open-ended question pre- and post-intervention.

Category	Sub-Category	Pre	Post	Example Pre-Intervention	Example Post-Intervention
Outlook	Attitude	17	28	Not worrying about small problems in life	I know I can live with it.
	Emotional self-awareness	1	2	Pulling myself out of that dark place [suicidality]	Seeing the psychiatrist for hypnosis to help my tinnitus and being able to talk with him about other stressful things
	Appreciation	7	2	I am grateful for my life	I enjoy my family and dogs.
	Perspective	4	2	Thankful that it is not louder	Thankful that it is not louder
	Empathy	17		I now have more compassion for people who have tinnitus	
	Resilience	4		I can beat it	
Personal development	Self-care	4		I take care of my health and food more	
	Self-awareness of capabilities	3	5	I am more aware of what I can handle	It makes me stronger in the face of adversity
	Self-control	1	1	The ability to cue my body and mind to relax on call is better	I am less of a risk taker
	Self-improvement	2		I applied to graduate school […] to study tinnitus	
	Motivation		1		I am working on my weight
Coping	Adaptation	9	14	I treat the tinnitus with white noise when sleeping	When I concentrate on things do not notice tinnitus
	Peaceful behavior		4		It brought me closer to meditation
	Spirituality	6		I view these physical trials as a positive thing from my lord and savior	
	Behavioral change	1	8		I am protecting my hearing by wearing earplugs
Support	Intimate relationships	5	1	It has made me realize how much friends and family care	I was able to tell my children that the sound of buzzing or cracking was our family’s normal
	Helping others	2		The chance to help others by helping to bring a solution about	
Treatment-related	Added benefit of treatment	11	29	I listen to more music	I am more aware of my surroundings.
	New relationships	1		I have met some really nice people in the tinnitus support group I am in	
	Behavior change	5	18	I am protecting my hearing by wearing earplugs	I am protecting my hearing by wearing earplugs
	Learned new techniques and skills		14		The sound enrichment and sound tolerance parts were very helpful for me
Disease-specific	Added benefit from tinnitus	5	8	I think it may actually help lull me to sleep	A contributing factor to getting a puppy…the puppy is adorable
	Direct benefit from tinnitus	2	3	I became more […] educated about my condition	Makes me focus on some things more

## Data Availability

The data that support the findings of this study are openly available in Figshare at http://doi.org/10.6084/m9.figshare.13681924 (accessed on 18 August 2024).
